# Supplementary material for: STING-dependent paracriny shapes apoptotic priming of breast tumors in response to anti-mitotic treatment
Source: Nat Commun. 2020 Jan 14;11:259. doi: 10.1038/s41467-019-13689-y (PMC6959316; doi:10.1038/s41467-019-13689-y)
Supplement: Supplementary file 1 — Supplementary Information [file 41467_2019_13689_MOESM1_ESM.pdf]

STING-dependent paracriny shapes apoptotic priming of breast tumors in response to anti-mitotic treatment

Lohard Steven[1,2], Bourgeois Nathalie[1,2,3], Maillet Laurent[1,2], Gautier Fabien[1,2,3], Fétiveau Aurélie[1,2], Lasla Hamza[2,3], Nguyen Frédérique[1,4], Vuillier Céline[1,2], Dumont Alison[1,2], Moreau-Aubry Agnès[1], Frapin Morgane[5], David Laurent[6,7], Loussouarn Delphine[8], Kerdraon Olivier[2,3], Campone Mario[1,2,3], Jézéquel Pascal[1,2,3], Juin Philippe P[1,2, 3, 9], Barillé-Nion Sophie[1,2, 9]

SUPPLEMENTARY FIGURES AND FIGURE LEGENDS

Supplementary Figure 1

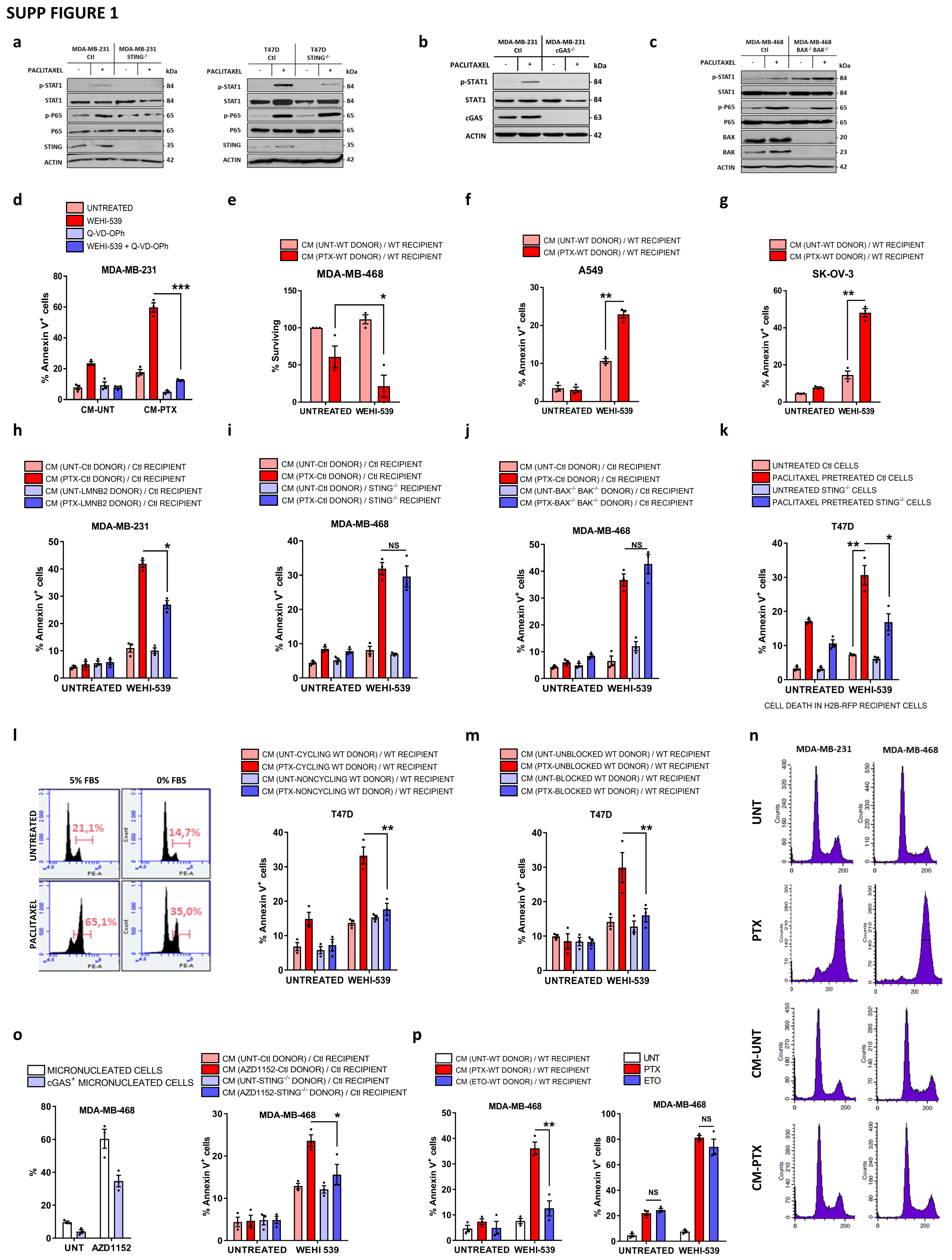

Supplementary Figure 1: Paclitaxel treatment triggers cGAS/STING signalling activation in breast cancer cells. (**a-c**) STAT1 and NF-κB signaling pathway immunoblot analysis in paclitaxel-treated or not, control or STING$^{-/-}$ (a) or cGAS$^{-/-}$(b) or BAX$^{-/-}$BAK$^{-/-}$(c) indicated breast cancer cells lines. (**d**) Annexin V assay in MDA-MB-231 cells treated by paclitaxel-induced or control CM plus WEHI-539 or not, in presence of the pancaspase inhibitor Q-VD-OPh or not. (**e**) Clonogenic assay using paclitaxel-induced or control CM in presence of WEHI-539 or not. (**f-g**) Same experiments as in (d) in A549 (f) or SK-OV-3 (g) cancer cell lines. (**h-j**) Same experiments as in (d) using LMNB2 overexpressing as donor (h), STING$^{-/-}$ recipient (i) or BAX$^{-/-}$BAK$^{-/-}$ donor (j) cells in the indicated cell lines. (**k**) 24h-paclitaxel-pretreated control or STING$^{-/-}$ T47D cells were cultured with untreated T47D H2B-RFP expressing cells. After 48h, co-cultures were treated or not with WEHI-539 for additional 48h and cell death was assessed in each cell population. (**l**) paclitaxel-treated or not, serum-deprived (non cycling) or not (cycling) T47D donor cells (cell cycle analysis on the left) were washed out to produce 48 h-CM that were applied to untreated corresponding cancer cells for 48h in presence or not of WEHI-539. Apoptotic index in recipient cells was assessed using Annexin-V staining (right). (**m**) Same experiment as in (l) with thymidine-blocked or control donor cells. (**n**) Cell cycle analysis in recipient control or paclitaxel-treated cells or after 48h-exposure to CM from untreated or paclitaxel-treated cells. (**o**) cGAS-positive micronuclei after treatment by the Aurora-B inhibitor AZD1152 in MDA-MB-468 cell line and Annexin V assay after cell exposure to AURKB inhibitor-induced CM in presence of WEHI-539 or not. (**p**) Annexin V assay after etoposide or paclitaxel-induced CM (left panel) or a direct 48h treatment on MDA-MB-468 cell line (right panel). Data were collected from n=3 independent experiments. Error bars indicate mean +/- SEM; two-sided paired *t*-test. The symbols correspond to a p-value inferior to *0.05, **0.01 and ***0.001. NS: Not significant.

SUPP FIGURE 2

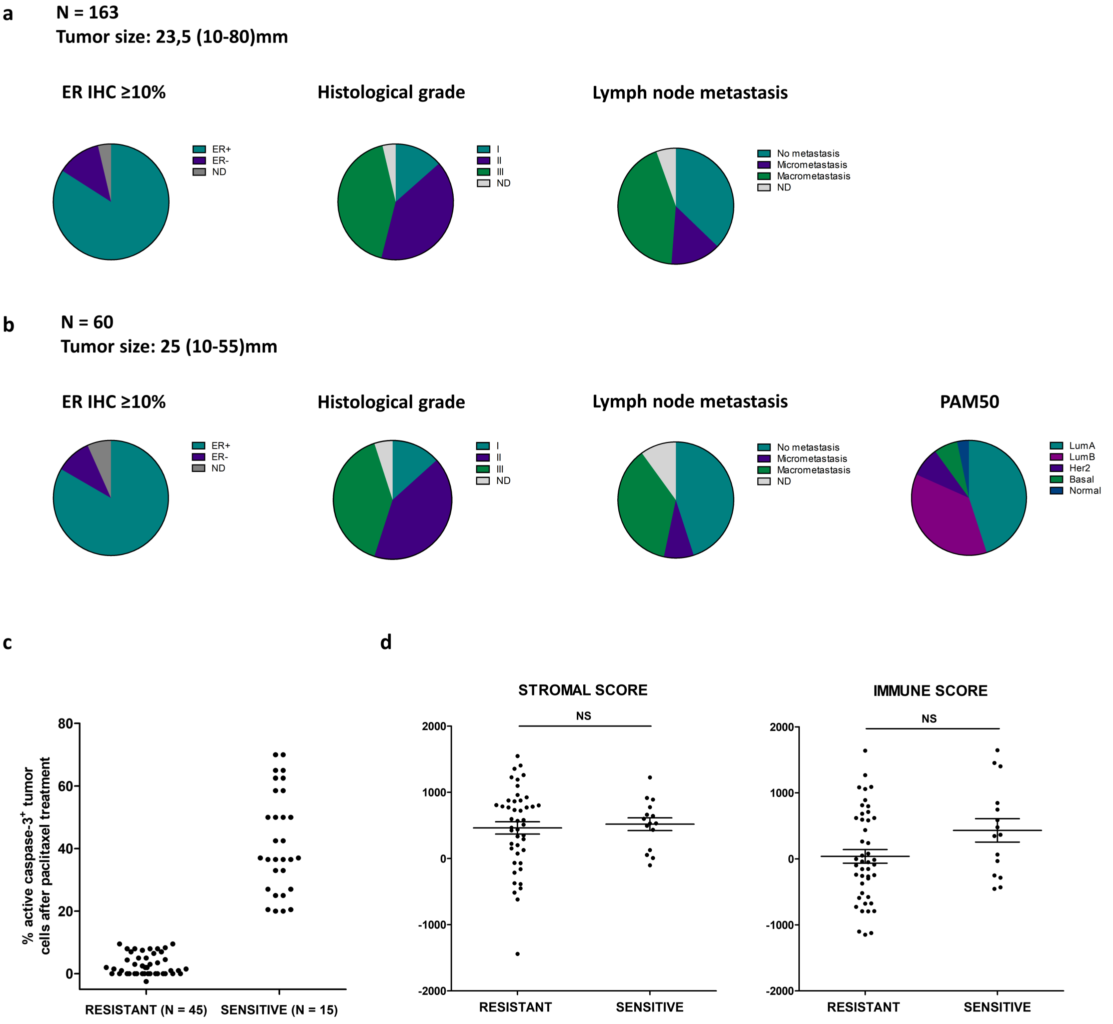

Supplementary Figure 2: Characteristics of breast tumors included in the study. (**a-b**) Tumor size, IHC ER status, histological grade, lymph node metastasis status and PAM50 (only for b) features of the cohort N=163 (n=163 tumors) (a) and of the cohort N=60 (n=60 tumors) (b). (**c**) Percentage of active caspase-3-positive cancer cells after paclitaxel treatment in the cohort N=60 (n=60 tumor). (**d**) Stromal and immune scores obtained from differential gene expression analysis based on tumor paclitaxel sensitivity (n=60 tumors). Error bars indicate mean +/- SEM; Two-sided unpaired *t*-test. NS=not significant

SUPP FIGURE 3

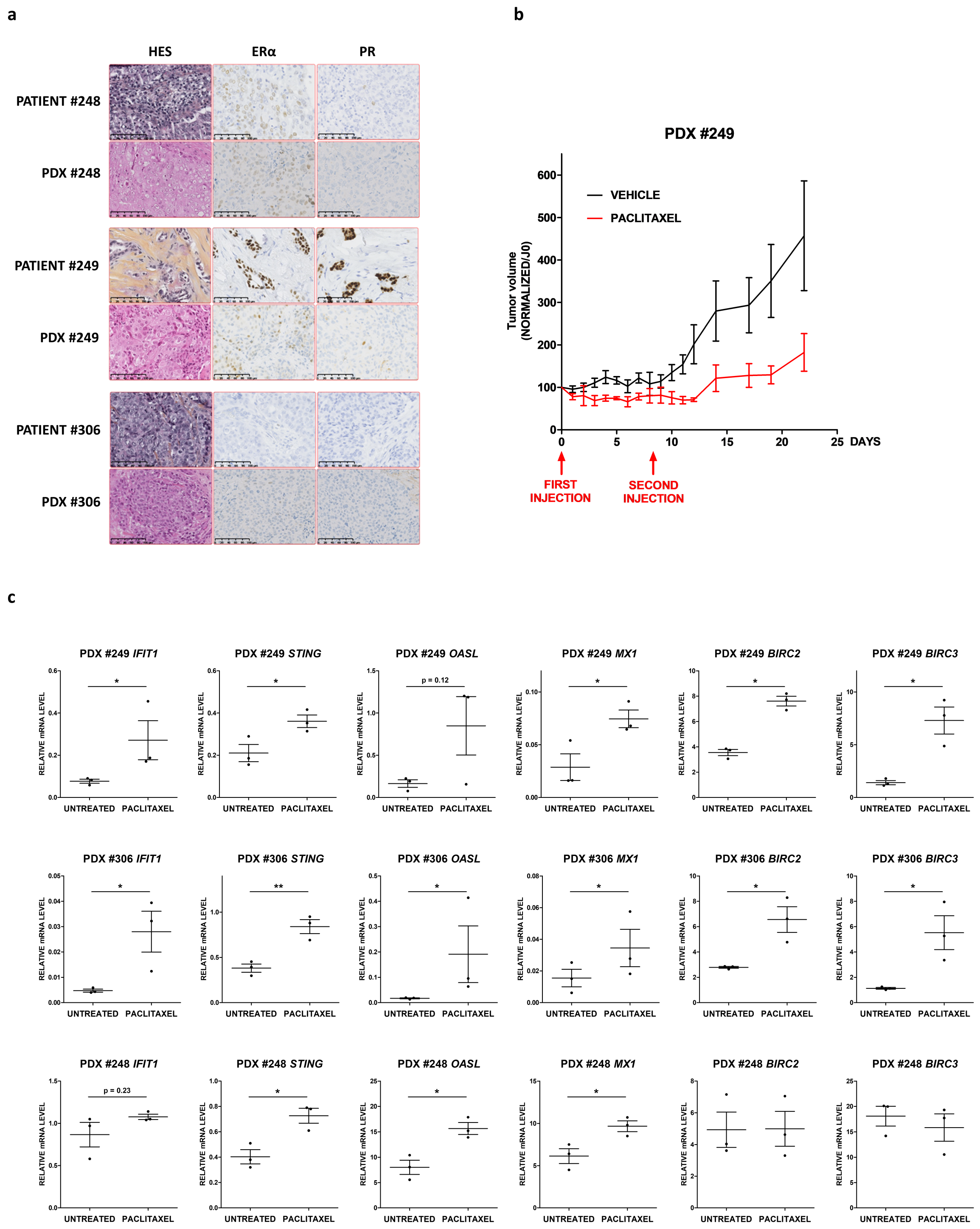

Supplementary Figure 3: Paclitaxel treatment triggers cGAS/STING signalling activation in breast cancer cells. (**a**) Histological features of breast tumors and corresponding PDX. (**b**) Paclitaxel antitumor effect on PDX #249 (n=5 mice per group). **(c)** qPCR analysis in PDX treated or not by paclitaxel for the indicated genes.

SUPP FIGURE 4

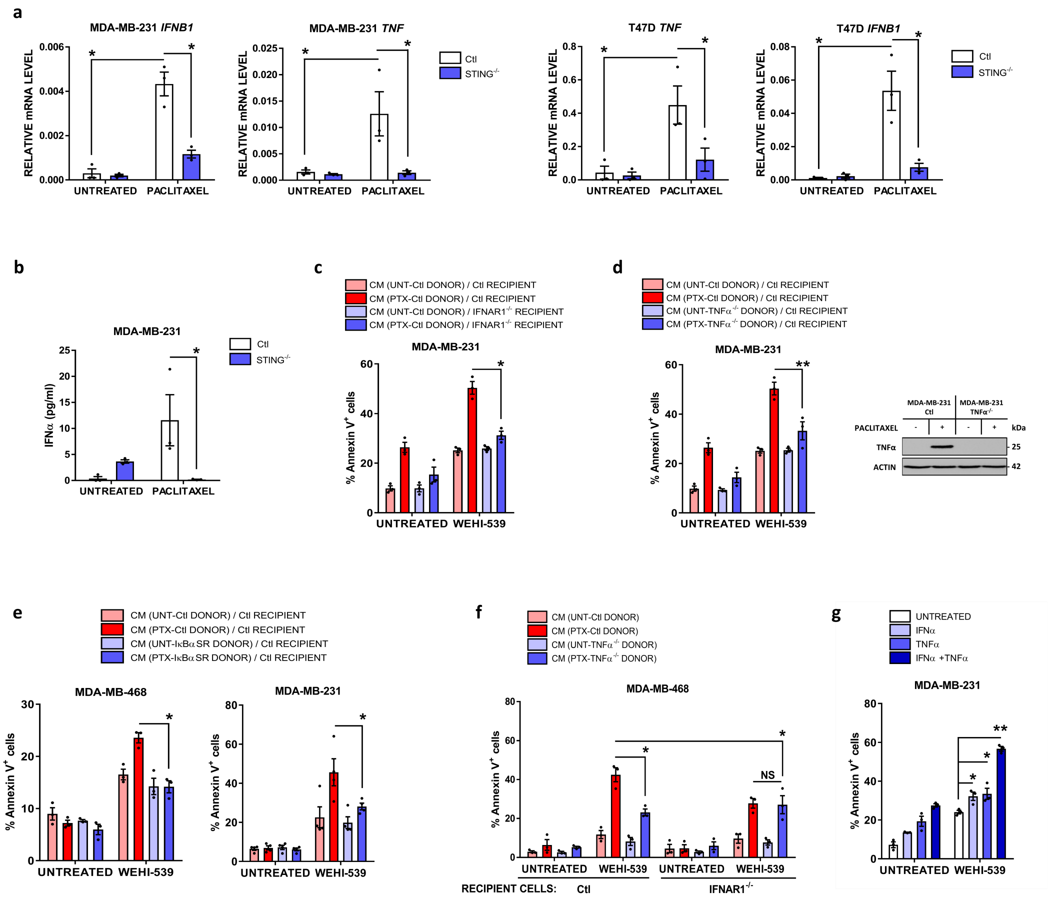

Supplementary Figure 4: Paclitaxel-induced paracrine apoptotic signalling relies on type I IFN and TNF pathways. (**a**) *TNF* and *IFNB1* qPCR analysis in paclitaxel-treated or not control or STING$^{-/-}$ MDA-MB-231 and T47D breast cancer cells lines. (**b**) IFNα production before and after paclitaxel treatment in control or STING$^{-/-}$ MDA-MB-231 cells. (**c-d**) Annexin V assay in MDA-MB-231 cells treated by paclitaxel-induced or control CM plus WEHI-539 or not using control or IFNAR1$^{-/-}$ recipient cells (c), and control or TNFα$^{-/-}$ donor cells (d). TNFα expression was detected by immunoblot analysis in monensin-treated cells 72h after 24h paclitaxel treatment or not (d, right panel). (**e**) Annexin-V assay as (c) using control or IκBαSR cells as donor indicated cells and IκBα immunoblot analysis. (**f**) Annexin-V assay as (c) using control or TNF$^{-/-}$ as donor cells and control or IFNAR1$^{-/-}$ MDA-MB-468 recipient cells. (**g**) Apoptotic effect of recombinant IFNα and/or TNFα on MDA-MB-231 cells treated or not with WEHI-539 during 48h. Data were collected from n=3 independent experiments. Error bars indicate mean +/- SEM; two-sided paired *t*-test. The symbols correspond to a p-value inferior to *0.05. NS=not significant.

SUPP FIGURE 5

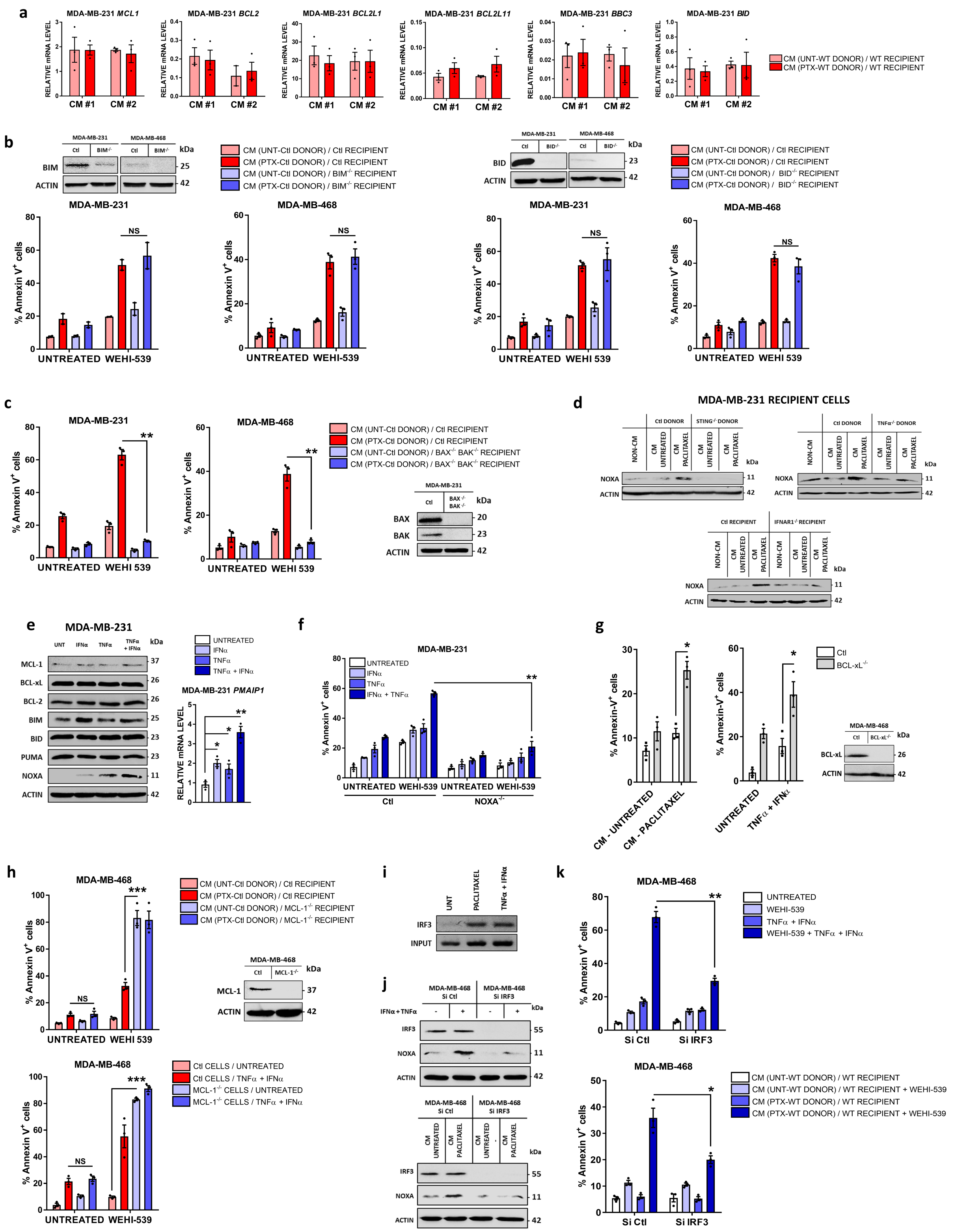

Supplementary Figure 5: Paclitaxel paracrine pro-apoptotic effect relies on STING/TNFα/type I IFN-dependent NOXA induction. (**a**) qPCR analysis in MDA-MB-231 recipient cells incubated for 48h with #1 or #2 CM consecutively produced during 48h. (**b-c**) Control donor cells treated for 24h by paclitaxel or not, were washed out to produce 48h-CM that were applied to control or BIM$^{-/-}$ (b, left panel), BID$^{-/-}$ (b, right panel) or double KO BAX$^{-/-}$ BAK$^{-/-}$ (**c**) corresponding cancer cells for 48h in presence or not of WEHI-539. Apoptotic index in recipient cells was assessed using Annexin-V staining. Analysis of BIM, BID, BAX and BAK expressions was realized by immunoblot. (**d**) NOXA immunoblot analysis in MDA-MB-231 recipient cells incubated for 48h with 48h-CM from control, STING$^{-/-}$ or TNFα$^{-/-}$ (upper panel) donor cells pretreated or not with paclitaxel and in control or IFNAR1$^{-/-}$ MDA-MB-231 recipient cells incubated for 48h with 48h-CM from control donor cells treated or not with paclitaxel (lower panel). (**e**) Immunoblot and *PMAIP1* qPCR analysis in MDA-MB-231 cells treated or not with recombinant TNFα and/or IFNα for 48h. (**f**) Proapoptotic effect of recombinant IFNα and/or TNFα in control or NOXA$^{-/-}$ MDA-MB-231 cells treated or not with WEHI-539 during 48h. (**g**) Control and BCL-xL$^{-/-}$ MDA-MB-468 cells were incubated for 48h with CM produced as experiment (b) or treated or not with recombinant IFNα and TNFα for 48h (right panel). Apoptotic index was assessed using Annexin-V staining. Analysis of BCL-xL expression was realized by immunoblot. (**h**) Same experiment as (g) using control and MCL-1$^{-/-}$ MDA-MB-468 cells treated or not with WEHI-539. (**i**) IRF3 and RelA binding to NOXA promoter by ChIP after paclitaxel or TNFα and IFNα 48h treatment in MDA-MB-468 cells. (**j-k**) NOXA immunoblot analysis (j) and Annexin V assay (k) in siRNA IRF3 knocked down MDA-MB-468 cells after treatment by TNFα and IFNα or after cell exposure to paclitaxel-treated or control IRF3 knocked-down or control cells corresponding CM for 48h in presence or not of WEHI-539. Data were collected from n=3 independent experiments. Error bars indicate mean +/- SEM; Two-sided paired *t*-test. The symbols correspond to a p-value inferior to *0.05, **0.01 and ***0.001. NS: Not significant.

SUPP FIGURE 6

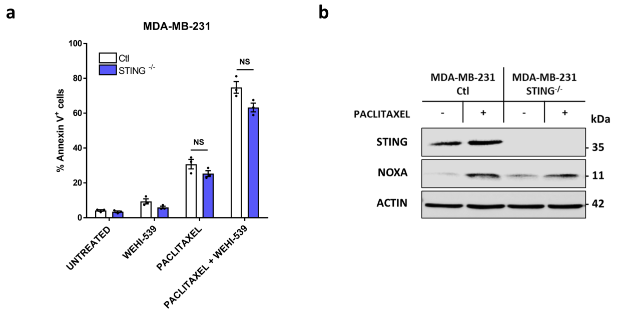

Supplementary Figure 6: Paclitaxel-induced NOXA expression in donor cells is STING-independent. (**a**) Annexin V assay in control and STING$^{-/-}$ MDA-MB-231 cells treated or not with paclitaxel for 24h, washed out for 48h and treated or not with WEHI-539 for additional 24h. (**b**) Immunoblot analysis in control and STING$^{-/-}$ MDA-MB-231 cells harvested 48h after 24h-paclitaxel treatment. Data were collected from n=3 independent experiments. Error bars indicate mean +/- SEM; Two-sided paired *t*-test. NS: Not significant.

SUPP FIGURE 7

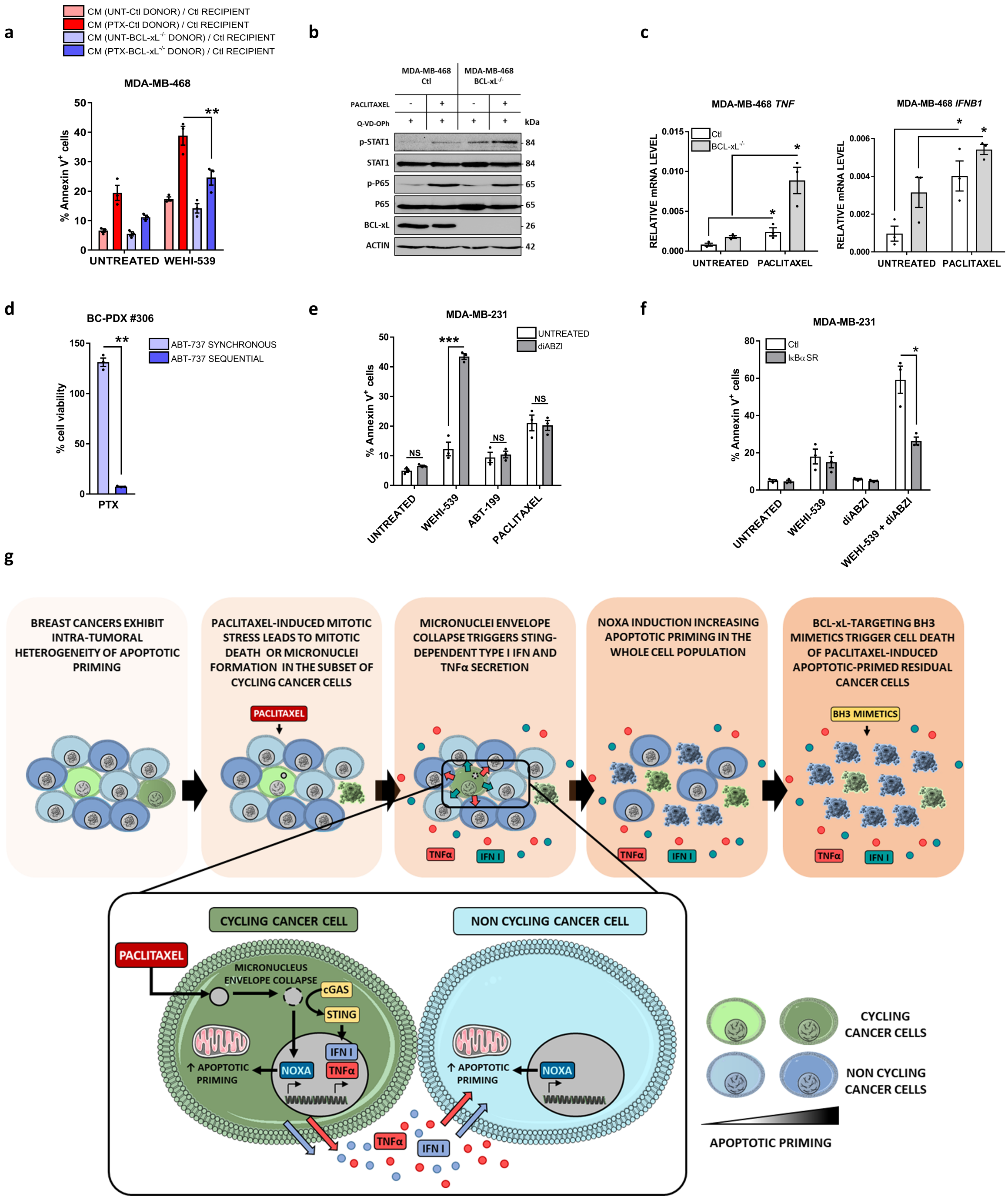

Supplementary Figure 7: BCL-xL in donor cells potentiates paclitaxel paracrine effect.

(**a**) 24h-paclitaxel-treated or not, control or BCL-xL$^{-/-}$ (donor) cells were washed out to produce 48h-CM that were applied to untreated (recipient) corresponding cancer cells for 48h in presence or not of the BH3 mimetic WEHI-539. Apoptotic index in recipient breast cancer cells was assessed using Annexin-V staining. (**b-c**) Immunoblot (b) and *TNF* and *IFNB1* qPCR analysis (c) in Q-VD-OPh (pan-caspase inhibitor-treated control or BCL-xL$^{-/-}$ MDA-MB-468 cells for 48h following a 24h-paclitaxel treatment or not. (**d**) Cell viability of the PDX#306-derived organoid after the 96h *ex vivo* synchronous paclitaxel and ABT-737 or paclitaxel plus 48h delayed ABT-737 treatment. (**e**) Annexin V assay in IκBαSR expressing MDA-MB-231 or control after 48h treatment with the STING agonist diABZI plus WEHI-539. (**f**) Same experiment as in (e) using MDA-MB-231 cells, treated by diABZI plus WEHI-539, ABT-199 or paclitaxel or not. (a-f) Data were collected from n=3 independent experiments. Error bars indicate mean +/- SEM; Two-sided paired *t*-test. The symbols correspond to a p-value inferior to *0.05, **0.01 and ***0.001. NS: Not significant. (**g**) Summary graphic of direct and indirect effects induced by paclitaxel treatment and sequential use of BCL-xL targeting BH3 mimetic treatment.

Supplementary Figure 8: Original scans of immunoblots related to Figures 1, 5, 6 and 7

UNPROCESSED SCANS OF WESTERN BLOT FROM FIGURE 1

a

p-STAT1

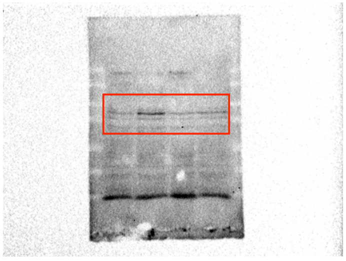

STAT1

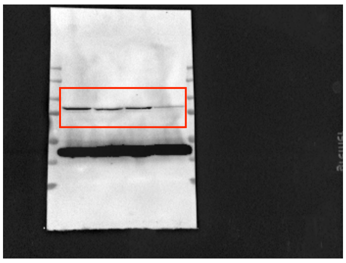

p-P65

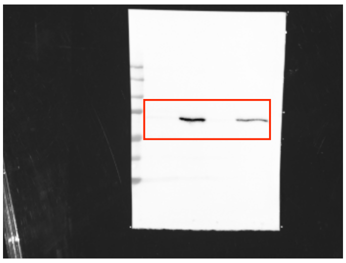

P65

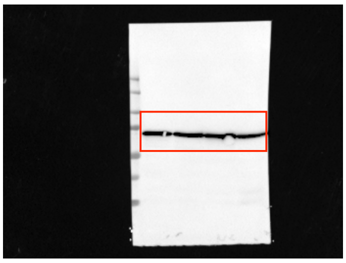

STING

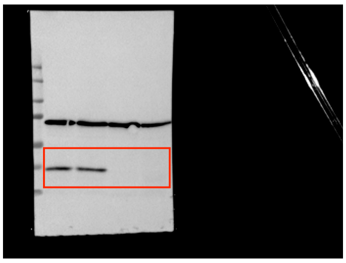

ACTIN

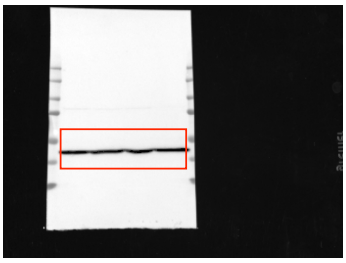

b

p-STAT1

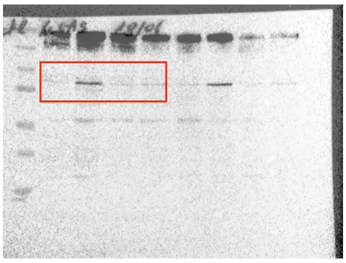

STAT1

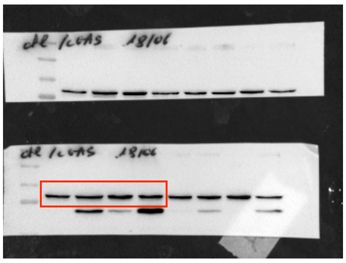

cGAS

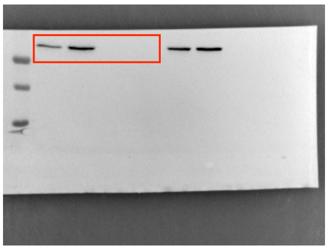

ACTIN

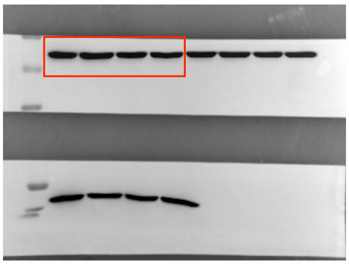

UNPROCESSED SCANS OF WESTERN BLOT FROM FIGURE 5

a

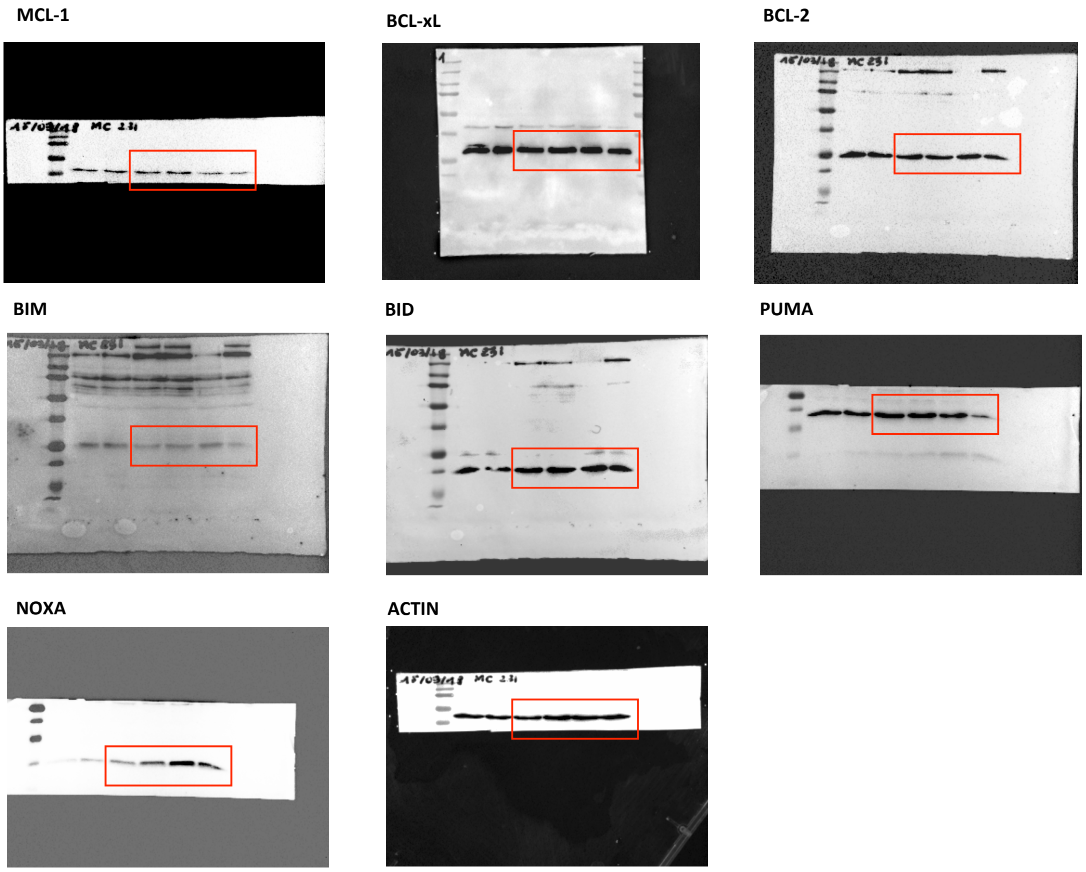

c

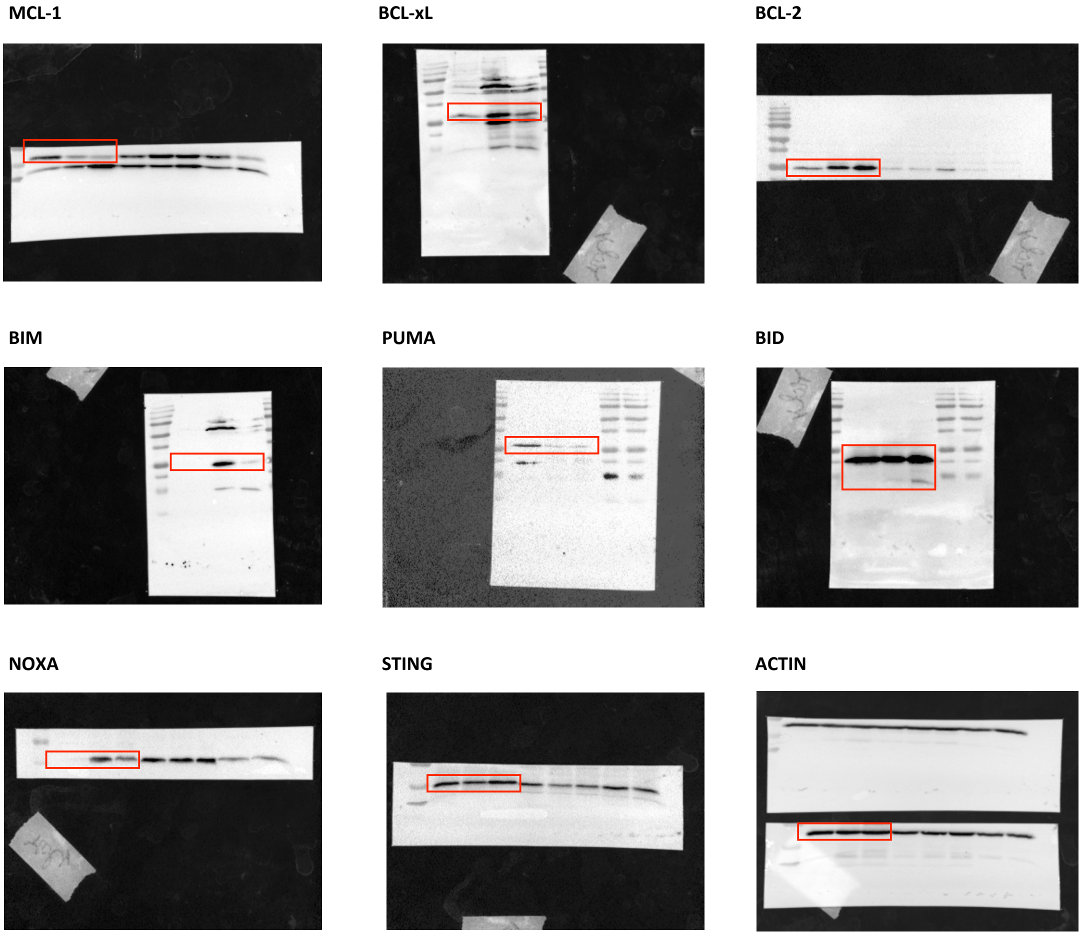

e

UPPER PANEL

NOXA

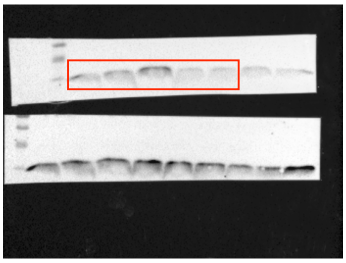

ACTIN

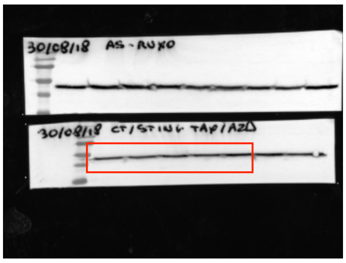

MIDDLE PANEL

NOXA

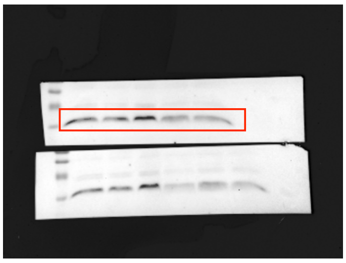

ACTIN

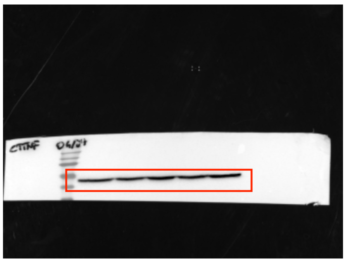

LOWER PANEL

NOXA

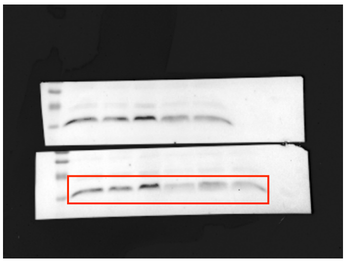

ACTIN

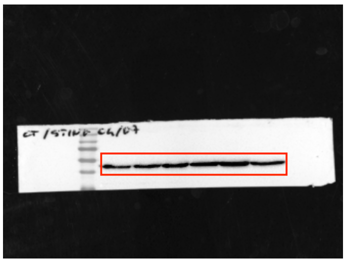

f

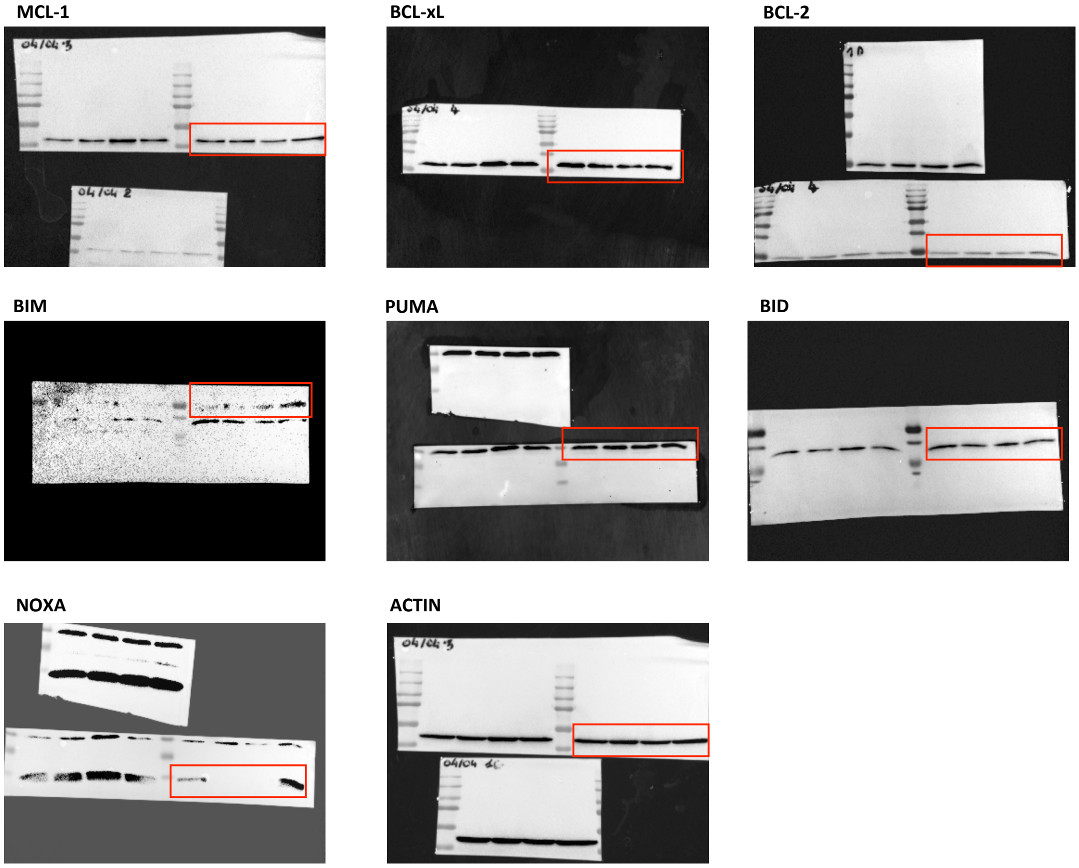

UNPROCESSED SCANS OF WESTERN BLOT FROM FIGURE 6

a

BCL-xL

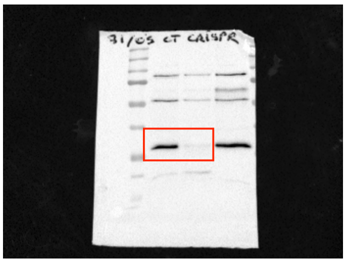

ACTIN

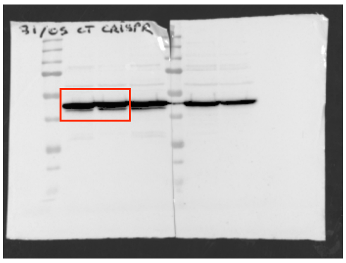

MCL-1

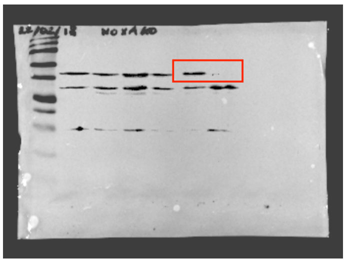

ACTIN

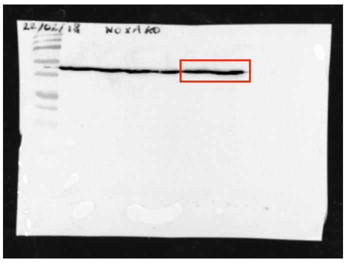

c

NOXA

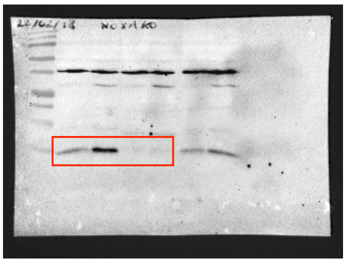

ACTIN

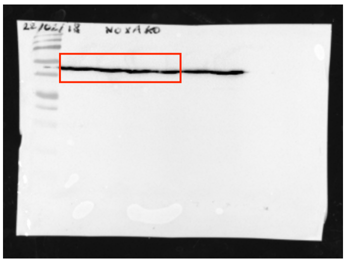

NOXA

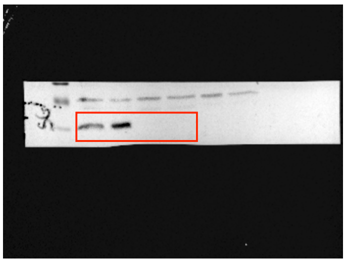

ACTIN

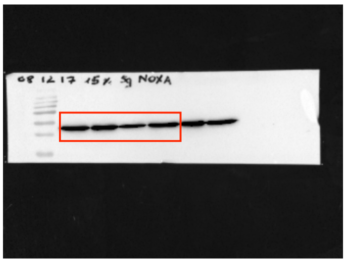

h

STING

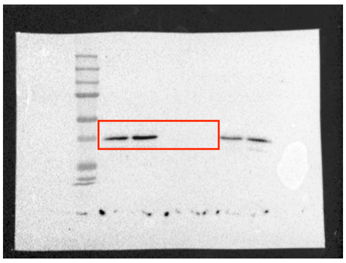

NOXA

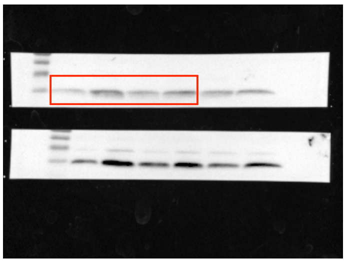

ACTIN

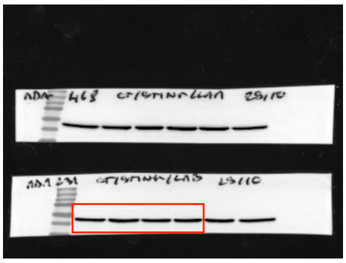

UNPROCESSED SCANS OF WESTERN BLOT FROM FIGURE 7

b

p-STAT1

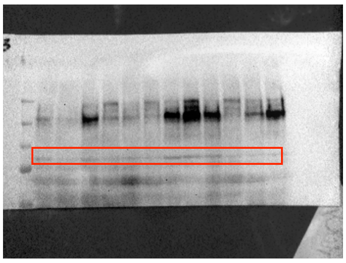

STAT1

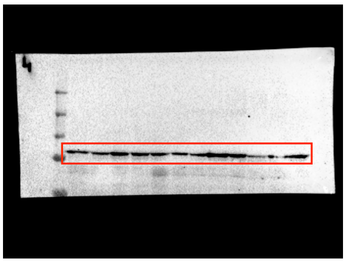

p-P65

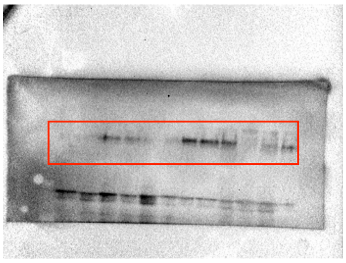

P65

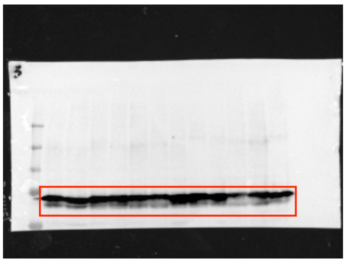

MCL-1

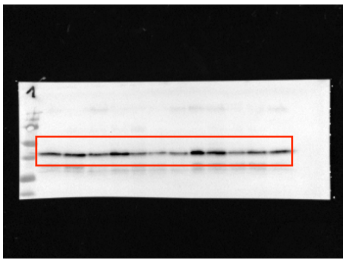

BCL-xL

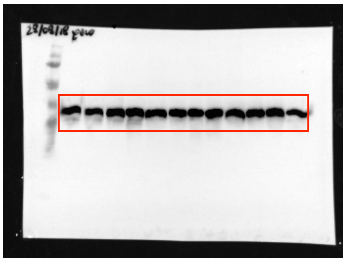

BIM

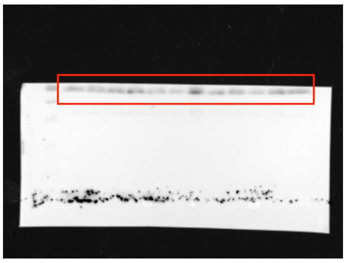

BID

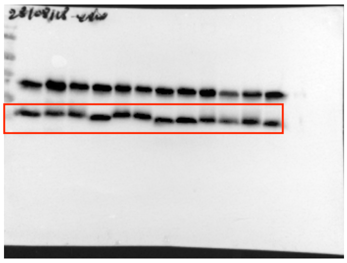

PUMA

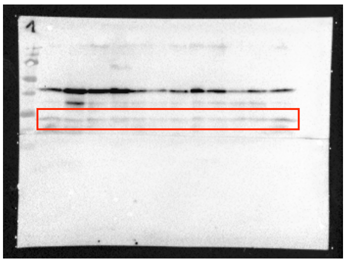

NOXA

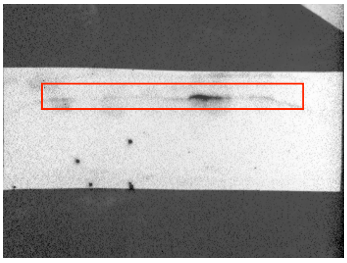

STING

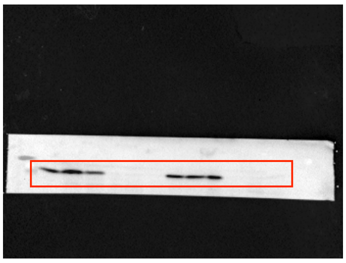

ACTIN

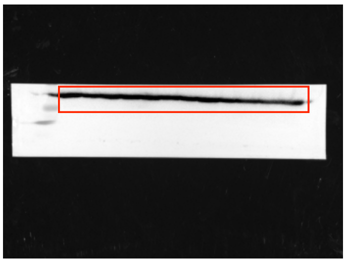

i

NOXA

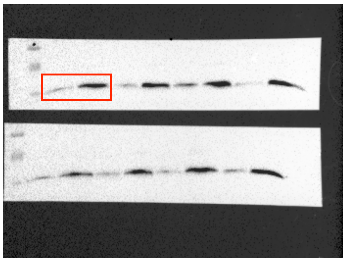

ACTIN

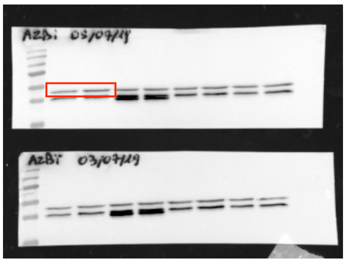

Supplementary Figure 9: Original scans of immunoblots related to Supplementary Figures 1, 5, 6 and 7

UNPROCESSED SCANS OF WESTERN BLOT FROM SUPPLEMENTARY FIGURE 1

a

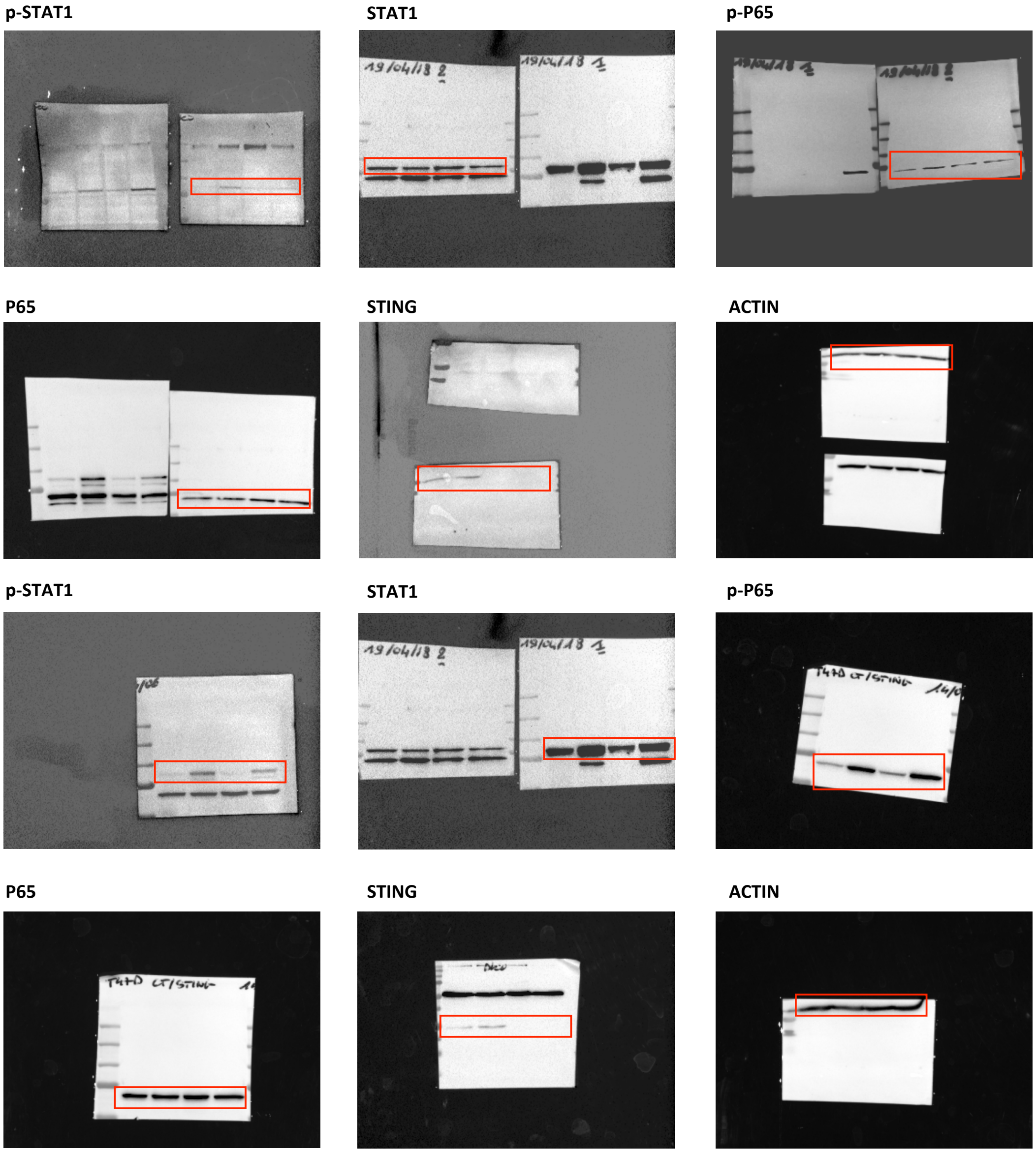

c

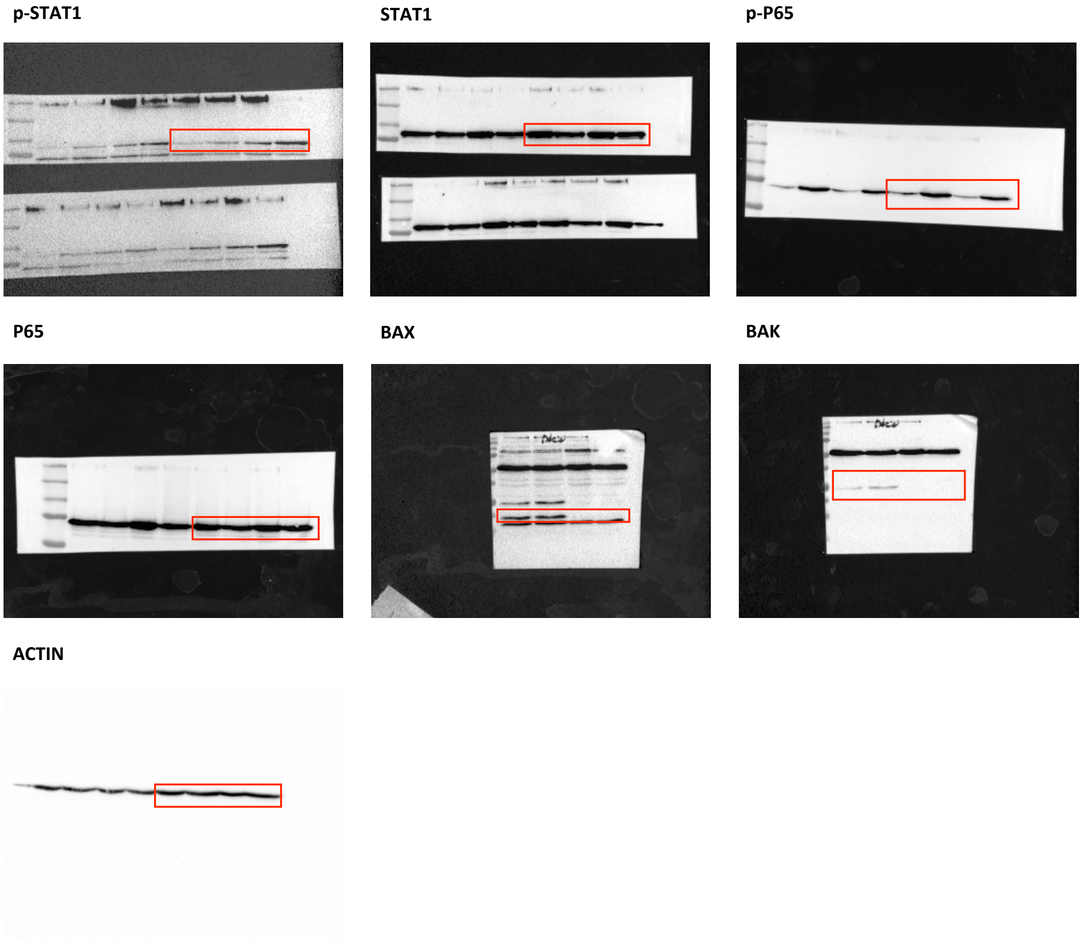

UNPROCESSED SCANS OF WESTERN BLOT FROM SUPPLEMENTARY FIGURE 5

f

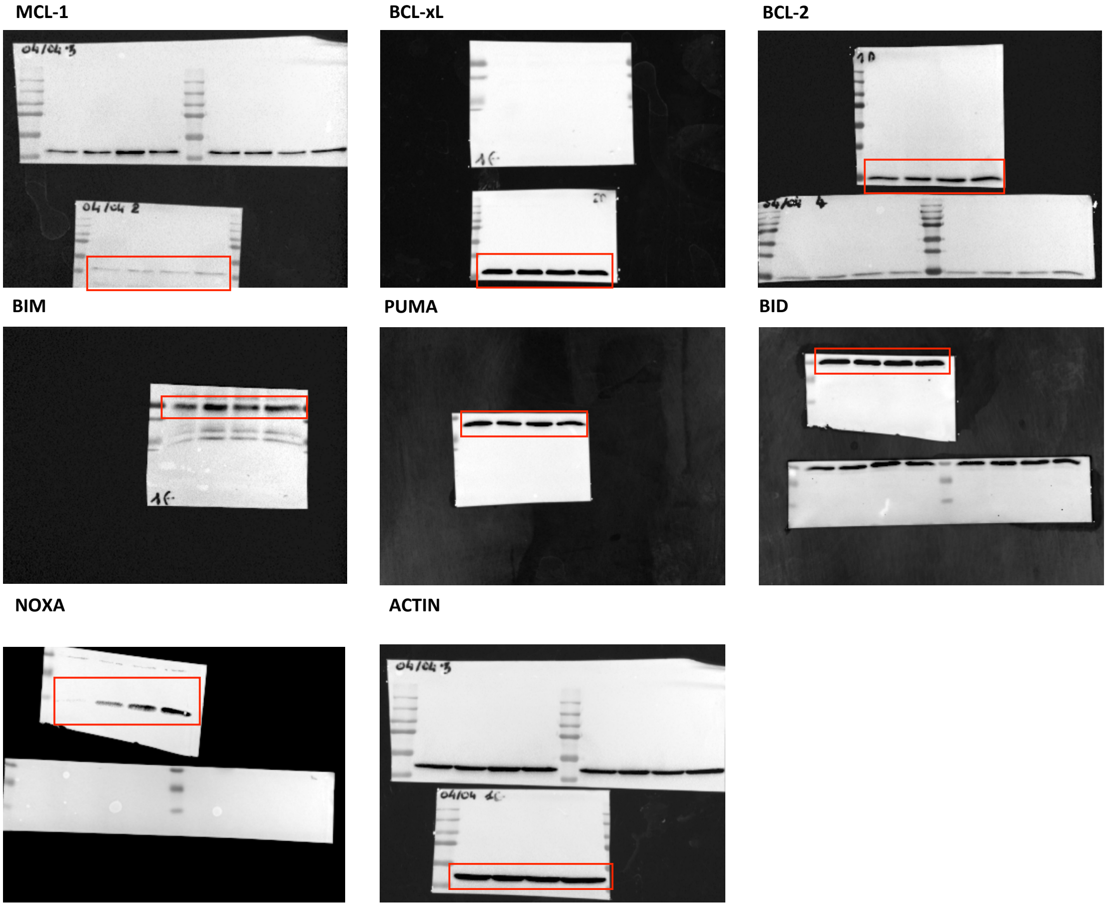

j

UPPER PANEL

IRF3

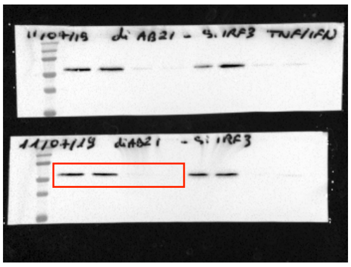

NOXA

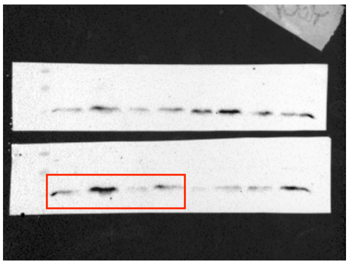

ACTIN

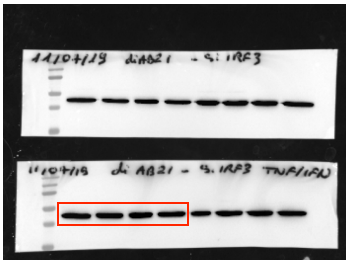

LOWER PANEL

IRF3

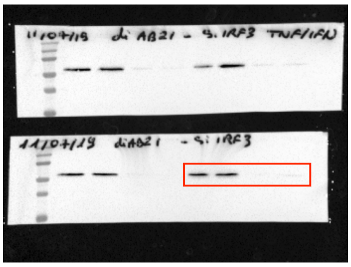

NOXA

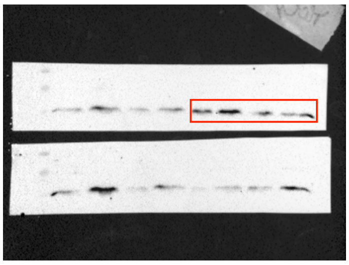

ACTIN

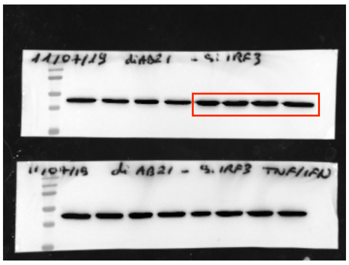

UNPROCESSED SCANS OF WESTERN BLOT FROM SUPPLEMENTARY FIGURE 6

b

NOXA

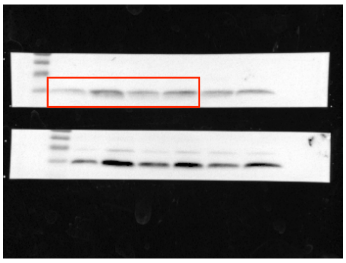

STING

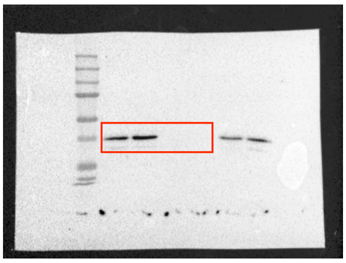

ACTIN

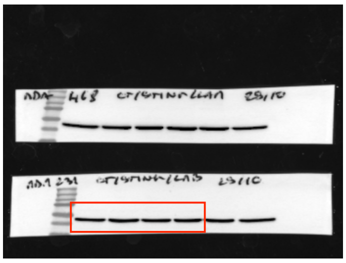

UNPROCESSED SCANS OF WESTERN BLOT FROM SUPPLEMENTARY FIGURE 7

b

p-STAT1

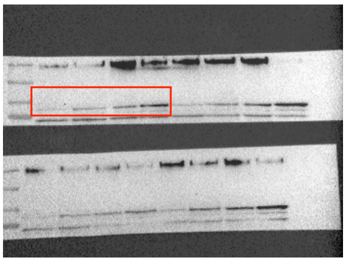

STAT1

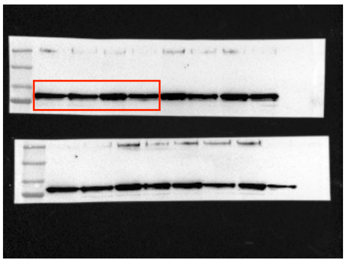

p-P65

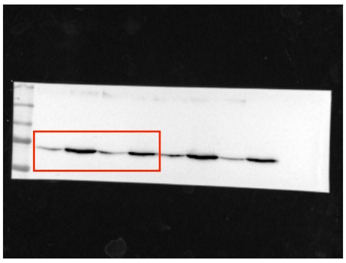

P65

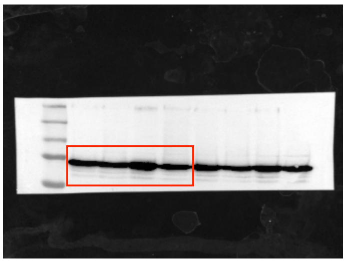

BCL-xL

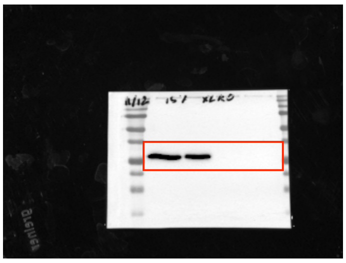

ACTIN

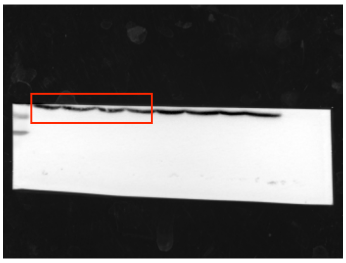

SUPPLEMENTARY METHODS

Gene expression signature scores calculation

Twelve gene expression signatures (GES) were selected for evaluation of different biological pathways and features. Five GES were for immune response dissection (interleukin-8 [IL-8], MHC-1, MHC-2, STAT1 and type I interferon [type I IFN]), 2 for metabolism evaluation (glycolysis and iron [iron regulatory gene signature: IRGS]), and 5 for critical biological pathways in cancer (chromosomal instability [CIN], mitochondrial oxidative phosphorylation [MITO/OXPHOS], proliferation, reactive stroma and wound response)[1-6].

GES scores were calculated for each patient using average expression or weighted average expression of combinations of gene or probe expressions.

	GES name	N° genes	N° probes	Statistics	Reference
Immune response	IL-8	-	4	AE	S1
	MHC-1	-	17	AE	S1
	MHC-2	-	14	AE	S1
	STAT1	-	14	AE	S1
	Type I IFN	-	14	AE	S1
Metabolims	Glycolysis	6	-	AE	S2
	IRGS	-	19	WAE	S3
Critical biological pathways in cancer	CIN	25	-	AE	S4
	MITO/OXPHOS	38	-	AE	S5
	Proliferation	-	47	AE	S1
	Reactive stroma	-	47	AE	S1
	Wound response	459	-	WAE	S6

GES: gene-expression signature; AE: average expression; IRGS: iron regulatory gene signature; WAE: weighted average expression; CIN: chromosomal instability; MITO/OXPHOS: mitochondrial oxidative phosphorylation

ChIP assay

For Chromatin immunoprecipitation (ChIP) assay, (15 × 10^6) MDA-MB-468 cells were treated or not for 24h with Paclitaxel (100nM) or a combination of recombinant IFNα (2000 UI.mL^{-1}) and TNFα (10 ng/ml). Then the assay was conducted following supplier's protocol (Active Motif, La Hulpe, Belgium) using anti-IRF3 or anti-RelA antibodies (Active Motif). PCR amplification of IFN[7] or NF-κB response elements (defined using JASPAR tool http://jaspar.genereg.net/ for the latter) corresponding to 5'agagattggaaaaggaaactt 3' (reverse strand) and 5' ggtacttcccc 3' (forward strand), in the *PMAIP1* gene respectively, was conducted before (input) and after ChIP assays with specific primers (forward 5' ctgggacccggagctttctc 3' and reverse 5' gggtggggagagaccagagg 3' and forward 5' cccttctcctcccacctcgt 3' and reverse 5' ccgcagacggcgttatgg 3') generating a 184 or 171-pb amplicons for IRF and NF-κB respectively.

Clonogenic assay

Cells were seeded at 100 cells into 6-well cell culture plates and incubated at 37 °C in a 5% CO2 atmo-sphere. Cells were then incubated with CM from paclitaxel-treated cells or not and with WEHI-539 or not for 14 days, washed twice with PBS, and stained with 0.1% crystal violet. The colonies were then washed twice with water, and colonies were counted .

References:

1. Karn, T. *et al*. Homogeneous datasets of triple negative breast cancers enable the identification of novel prognostic and predictive signatures. *PLoS One* **6**, e28403 (2011).

2. Hu, Z. *et al*. A compact VEGF signature associated with distant metastases and poor outcomes. *BMC Med*. **7**, 9 (2009).

3. Miller, L.D. *et al*. An iron regulatory gene signature predicts outcome in breast cancer. *Cancer Res.* **71**, 6728-6737 (2011).

4. Carter, S.L., Eklund, A.C., Kohane, I.S., Harris, L.N. & Szallasi, Z. A signature of chromosomal instability inferred from gene expression profiles predicts clinical outcome in multiple human cancers. *Nat. Genet.* **9**, 1043-1048 (2006).

5. Whitaker-Menezes, D. *et al*. Hyperactivation of oxidative mitochondrial metabolism in epithelial cancer cells in situ: visualizing the therapeutic effects of metformin in tumor tissue. *Cell Cycle* **23**, 4047-4064 (2011).

6. Chang, H.Y. *et al*. Gene expression signature of fibroblast serum response predicts human cancer progression: similarities between tumors and wounds. *PLoS Biol.* **2**, E7 (2004).

7. Lallemand, C., Blanchard B., Palmieri M., Lebon P., May E., & Tovey, M.G. Single-stranded RNA viruses inactivate the transcriptional activity of p53 but induce NOXA-dependent apoptosis via post-translational modifications of IRF-1, IRF-3and CREB. *Oncogene*. **26**, 328–338 (2007).
